# Detecting and Quantifying Polyhaloaromatic Environmental Pollutants by Chemiluminescence-Based Analytical Method

**DOI:** 10.3390/molecules26113365

**Published:** 2021-06-02

**Authors:** Ben-Zhan Zhu, Miao Tang, Chun-Hua Huang, Li Mao

**Affiliations:** 1 State Key Laboratory of Environmental Chemistry and Ecotoxicology, Research Center for Eco-Environmental Sciences, Chinese Academy of Sciences, Beijing 100085, China; miaotang_st@rcees.ac.cn (M.T.); chhuang@rcees.ac.cn (C.-H.H.); 2 University of Chinese Academy of Sciences, Beijing 100049, China

**Keywords:** polyhaloaromatic compounds, chemiluminescence, analytical method, Fenton system, hydroxyl radicals

## Abstract

Polyhaloaromatic compounds (XAr) are ubiquitous and recalcitrant in the environment. They are potentially carcinogenic to organisms and may induce serious risks to the ecosystem, raising increasing public concern. Therefore, it is important to detect and quantify these ubiquitous XAr in the environment, and to monitor their degradation kinetics during the treatment of these recalcitrant pollutants. We have previously found that unprecedented intrinsic chemiluminescence (CL) can be produced by a haloquinones/H_2_O_2_ system, a newly-found ^●^OH-generating system different from the classic Fenton system. Recently, we found that the degradation of priority pollutant pentachlorophenol by the classic Fe(II)-Fenton system could produce intrinsic CL, which was mainly dependent on the generation of chloroquinone intermediates. Analogous effects were observed for all nineteen chlorophenols, other halophenols and several classes of XAr, and a novel, rapid and sensitive CL-based analytical method was developed to detect these XAr and monitor their degradation kinetics. Interestingly, for those XAr with halohydroxyl quinoid structure, a Co(II)-mediated Fenton-like system could induce a stronger CL emission and higher degradation, probably due to site-specific generation of highly-effective ^●^OH. These findings may have broad chemical and environmental implications for future studies, which would be helpful for developing new analytical methods and technologies to investigate those ubiquitous XAr.

## 1. Introduction

### 1.1. Polyhaloaromatics (XAr) and Their Toxicity

Polyhaloaromatic compounds (XAr) have been found world-wide in pesticides, pharmaceuticals, flame retardants and personal care products [1,2,3,4]. Most of these compounds are persistent and widely existing in the environment because of their recalcitrant properties in the soil and water. More importantly, not only the oxidative DNA damage, but also protein and DNA adducts may be induced by these XAr compounds in vitro and in vivo systems [5,6,7,8,9], which possibly makes them carcinogenic to mammalian organisms [10,11]. One typical group of XAr are polyhalophenols, some of which, such as 2,4,6-trichlorophenol and pentachlorophenol (PCP, the widely-used wood preservative) have been classified as priority pollutants by the U.S. Environmental Protection Agency (US-EPA) [12]. PCP was also classified as a group I human carcinogen by the International Agency for Research on Cancer (IARC) [13]. PCP is potentially carcinogenic to mammals. Hepatocellular carcinomas and hemangiosarcomas were observed from B6C3F1 mice under exposure to PCP [14]. In individuals with occupational exposure to PCP, malignant lymphoma and leukemia in humans were also found to relate to PCP [15].

### 1.2. The Detection of XAr

The widespread distribution and highly-toxic nature, together with the recalcitrant and carcinogenic characteristics of these XAr, have raised public concerns about their potential risks to human health and ecosystems [4,16,17,18,19,20,21]. Therefore, detecting and quantifying these widespread polyhaloaromatic pollutants or pharmaceutics in the environment is crucial. The traditional analytical methods used to detect XAr, such as UV−Vis spectrophotometry, high-performance liquid chromatography (HPLC) and gas chromatography (GC) [22,23], usually have many shortcomings: such as low sensitivity, time-consuming, requiring sample pretreatment, expensive apparatus and complicated operation. Therefore, a sensitive, simple, low-cost and effective analytical method to detect and quantify the ubiquitous XAr is urgently needed.

Chemiluminescence (CL) is well regarded as a kind of light emission from complicated chemical reactions, during which high-energy excited-states can be generated and energy is released [24,25,26,27]. Since the CL intensity depends on the rate of the chemical reactions, it can be used to detect and quantify the specific compounds that determine the generation of CL emission. For the CL-based analytical method, which exhibits the excellent properties of relatively simple, rapid, sensitive, without complicated pretreatment [28], has been widely used as the analytical method in environmental analysis, clinical diagnosis and food safety monitoring [29,30,31]. So if the CL analytical method can be successfully applied to detect and quantify the highly-toxic XAr, it will be significant for the degradation and pollution control of these persistent and recalcitrant substances.

### 1.3. The Degradation and Treatment of XAr

A variety of methods and technologies have been used to degrade and treat recalcitrant XAr in the environment, including enzymatic biodegradation, physical adsorption and chemically advanced oxidation. Among them, advanced oxidation processes (AOPs) have been considered to be the most widely-used means for degrading and treating XAr, mainly because they are highly-effective and environmentally green [2,32,33]. Several alternative AOPs, such as Fenton and Fenton-like oxidation [17,34], UV-photolysis [35], and ozonation [36], have also been developed to effectively degrade and treat the recalcitrant XAr. In these green AOP systems, the most reactive intermediate for degradation is the strong oxidative radical species hydroxyl radical (^●^OH) [32].

### 1.4. Unprecedented ^●^OH Generation and CL Emission Can Be Produced from H_2_O_2_ and Polyhaloquinones, the Carcinogenic Metabolites of XAr

The most well-known pathway for ^●^OH generation is through the classic Fenton or Fenton-like reactions mediated by reactive transition metal ions [37,38]. We recently found an unprecedented metal-independent ^●^OH-generating system: polyhaloquinones and H_2_O_2_, and the molecular mechanism of typical nucleophilic substitution coupling with homolytic decomposition for ^●^OH generation was proposed [3,39,40,41,42,43,44,45,46,47]. More interestingly, an unexpected intrinsic CL emission can also be produced in this novel ^●^OH-generating system, which was found to be specifically dependent on ^●^OH production [44,48,49,50,51]. Taking the reaction of tetrachloro-1,4-benzoquinone (TCBQ, a carcinogenic quinone metabolite of PCP) with H_2_O_2_ as an example, a typical two-step CL emission can be clearly observed, which is directly dependent on the two-step generating processes of ^●^OH [44]. Moreover, not only for TCBQ, but also for other polyhaloquinones, such as other chloroquinones, fluoroquinones, bromoquinones and halonaphthoquinones, similar intrinsic ^●^OH-dependent CL was produced. These results revealed an unprecedented ^●^OH-generating and CL-producing system: polyhaloquinones (XQs) and H_2_O_2_.

### 1.5. The Goal of This Paper

It has been previously known that a variety of XAr, such as PCP, can be chemically-degraded into haloquinones during the AOPs with the generation and involvement of ^●^OH [17,18,19,52,53,54]. Then we wonder whether ^●^OH-dependent CL emission can also be generated in the degradation of PCP mediated by these AOPs. In addition, as one of the important final products of TCBQ after the interaction with H_2_O_2_, 2,5-dichloro-3,6-dihydroxyl-1,4-benzoquinone (DDBQ, an inert halohydroxyl quinoid compound) would not react with H_2_O_2_ to generate ^●^OH and produce CL [44], but it is not clear whether the addition of extra ^●^OH will induce the production of CL emission. Therefore, in order to answer the above questions, the following issues were addressed in a series of our recent studies: (1) Can intrinsic CL be generated from the degradation of precursors XAr (such as PCP and other chlorophenols) mediated by AOPs involving ^●^OH-generation? (2) If so, what is the potential molecular mechanism for the CL emission, and is there any potential structure−activity relationship (SAR) between CL emission and the structures of XAr? (3) Do ^●^OH-generating systems induce the inert halohydroxyl quinoid compounds to generate CL? (4) If so, what is the role of the typical bidentate coordination sites on the structures of halohydroxyl quinoid compounds, and is the CL generated from them different to that from other XAr? (5) Can we develop a sensitive and selective CL-based analytical method for the detection and quantification of XAr? (6) What are the potential chemical and environmental implications?

## 2. Chemiluminescence-Based Analytical Methods Induced by Fe(II)-Fenton System for the Detection of XAr

### 2.1. Intrinsic ^●^OH-Dependent CL Emission Can Be Generated from the Degradation of the Priority Pollutant PCP in Fe(II)-Fenton System

**Figure 1:**
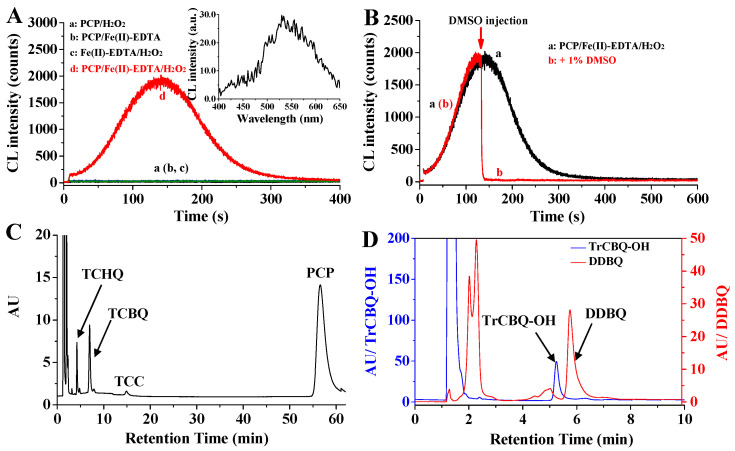
Intrinsic ^●^OH-dependent CL was generated from PCP degradation mediated by Fe(II)-Fenton system, during which chloroquinones were formed as the critical intermediates [48]. (**A**) Intrinsic CL emission was produced by PCP/Fe(II)-Fenton system; (**B**) ^●^OH scavenger DMSO can markedly inhibit CL emission; (**C**,**D**) several chloroquinone intermediates were generated from PCP during the CL emission. The CL measurement (**A**,**B**) was conducted through an ultraweak CL analyzer, with the CL signal recorded at a time interval of 0.1 s. Both the instant CL intensity and the intensity of total CL emission were recorded. The separation and identification of chloroquinone intermediates (**C**,**D**) generated from PCP degradation was conducted by HPLC analysis. For detailed experimental procedure, please refer to our previous study [48]. Fe(II)-EDTA, 1mM; H_2_O_2_, 100 mM. For (**A**,**B**) PCP, 20 μM; for (**C**,**D**) PCP, 1 mM. Copyright © 2015, American Chemical Society.

We have previously known that the reaction between TCBQ and H_2_O_2_ could unexpectedly produce highly-reactive ^●^OH and specific ^●^OH-dependent CL [44]. Moreover, PCP could be chemically degraded and converted to TCBQ in the classic ^●^OH-generating Fe(II)-Fenton system [52,54]. Then, we wanted to know whether CL could be generated in the PCP degradation process mediated by AOPs composed of the classic Fe(II)-Fenton system. As expected, neither ^●^OH nor CL emission was detected when incubating PCP with H_2_O_2_, whereas a remarkable CL emission (510−580 nm) was generated when extra ^●^OH was introduced by adding Fe(II)-EDTA (Fe(II)-ethylenediamine tetraacetic acid, a classic Fenton reagent) (Figure 1A), indicating that the degradation of PCP mediated by ^●^OH-generating Fe(II)-Fenton system indeed produce intrinsic CL emissions [48].

**Figure 2:**
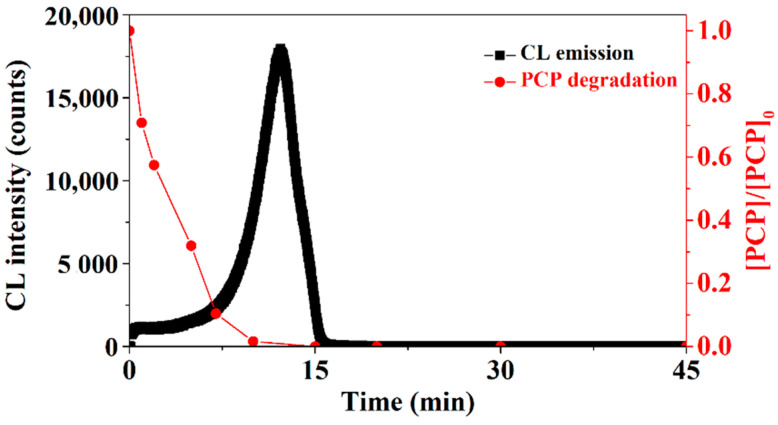
The CL emission from PCP/Fenton system correlated well with PCP degradation [48]. The CL emission was measured by an ultraweak CL analyzer, with the CL signal recorded at a time interval of 0.1s. The kinetics of PCP degradation was monitored by HPLC analysis. For detailed experimental procedure, please refer to our previous study [48]. PCP, 1 mM; Fe(II)−EDTA, 3 mM; H_2_O_2_, 300 mM. Copyright © 2015, American Chemical Society.

Interestingly, similar to the CL generated from TCBQ/H_2_O_2_, the CL derived from PCP/Fe(II)-Fenton system was also directly dependent on ^●^OH generation, as shown by the following line of evidence [48]: (1) The CL emission from the PCP/Fe(II)-Fenton system was significantly inhibited by dimethyl sulfoxide (DMSO), a typical ^●^OH scavenger (Figure 1B); (2) both the yields of ^●^OH and the intensity of CL emission increased with the increasing dosage of Fenton reagents; (3) CL was also produced from the other widely known ^●^OH-generating Fenton agent Fe(II)-NTA (nitrilotriacetic acid) [55], and a good correlation was observed between the CL emission and the kinetics of ^●^OH formation.

Previous studies have reported that the degradation of chlorophenol could produce chloroquinone as intermediates [52,54]. Considering that obvious CL could be generated from the interaction of TCBQ (or other haloquinones) with H_2_O_2_, we hypothesized that the critical species initiating the CL emission from PCP/Fe(II)-Fenton system might be such chloroquinone intermediates. As expected, five transient chloroquinone intermediates were identified (Figure 1C,D), they were TCBQ, tetrachloro-1,4-hydroquinone (TCHQ), tetrachloro-1,2-hydroquinone (tetrachlorocatechol, TCC), trichlorohydroxyl-1,4-benzoquine (TrCBQ-OH) and 2,5-dichloro-3,6-dihydroxyl-1,4-benzoquinone (DDBQ) [48]. It should be noted that besides DDBQ, the other four chloroquinones could undergo interactions with H_2_O_2_ to generate CL, and the addition of an extra ^●^OH-generating Fenton reagent can markedly enhance CL emission. These results verify that the CL production from PCP/Fe(II)-Fenton systems is originated from the generation of chloroquinones.

**Scheme 1:**
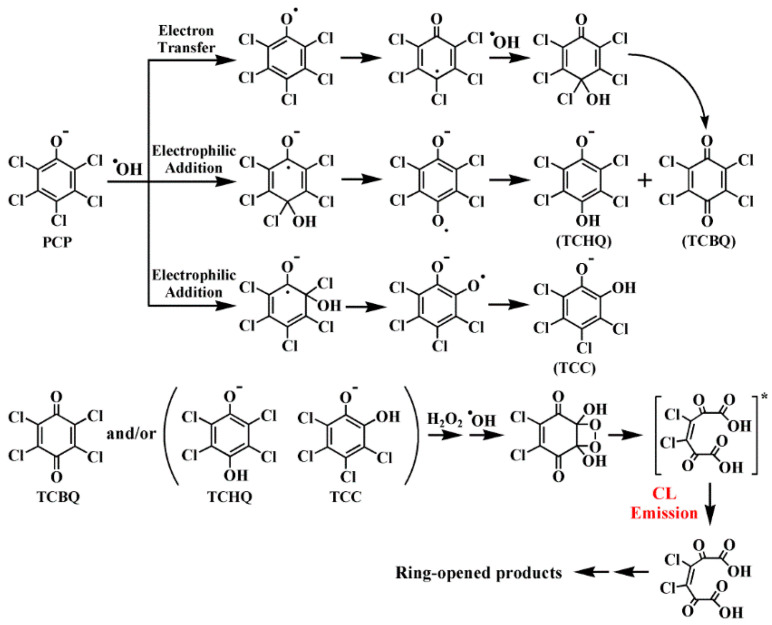
The molecular mechanism for the unexpected ^●^OH-dependent CL emission from the degradation of PCP mediated by Fe(II)-Fenton system [48]. The classic Fenton system could produce large amounts of reactive ^●^OH, which further attacks PCP via electrophilic addition and/or electron transfer pathways, forming pentachlorophenoxyl radical and tetrachlorosemiquinone radicals. The latter radicals then convert to tetrachloroquinones, which further react with H_2_O_2_ to produce high-energy quinone-1,2-dioxetanes, and finally emit the intrinsic ^●^OH-dependent CL as reported before. Copyright © 2015, American Chemical Society.

On the basis of our previously discovered mechanism for CL generation from the TCBQ/H_2_O_2_ system [44], and together with the above findings, we proposed the underlying molecular mechanism for ^●^OH-dependent CL emission from the degradation of PCP mediated by the classic ^●^OH-generating Fe(II)-Fenton system (Scheme 1) [48]:

Since remarkable CL emission can be unambiguously generated from PCP/Fe(II)-Fenton system and increasing the concentration of Fenton reagents can enhance CL, these suggest that it is possible to develop an undiscovered novel CL-based analytical method for the detection and quantification of PCP. Further studies proved it was indeed the case [48]: the unique CL-generating property of PCP was used to develop a novel analytical method for detecting and measuring trace amounts of PCP, and it was found that the LOD (limit of detection) value was 1.8 ppb and the linear range (LR) was 2.6–18,620 ppb for PCP as detected by this CL method. Both the LOD and LR values are lower than the concentration of PCP (40 ppb) in the body fluids of people under non-occupational exposure, and much lower than PCP (19,580 ppb) concentration under occupational exposure [3]. Interestingly, the kinetics of CL emission was found to correlate well with the kinetics of PCP degradation: when PCP degradation achieved the maximum, CL emission was no longer observed (Figure 2). These results indicate that this novel CL-based analytical method could also be used to monitor the degradation kinetics of PCP.

### 2.2. Analogous ^●^OH-Dependent CL Emission from the Degradation of All 19 Chlorophenols and the Underlying Structure−Activity Relationship

**Figure 3:**
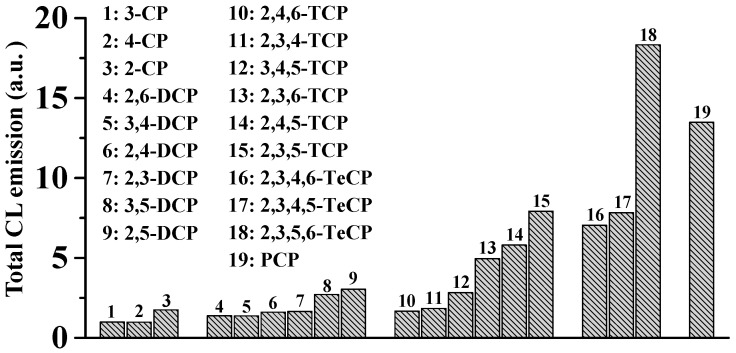
Intrinsic ^●^OH-dependent CL were also generated from all 19 chlorophenols in AOPs mediated by Fe(II)-Fenton system [48]. The CL emission was measured by an ultraweak CL analyzer, and the intensity of total CL emission was recorded. CPs, 10 μM; Fe(II)–EDTA, 1 mM; H_2_O_2_, 100 mM. Copyright © 2015, American Chemical Society.

The above findings that remarkable CL emission can be produced from the PCP/Fe(II)-Fenton system suggest that CL may also be generated from the interactions of other chlorophenols (CPs) with the classic Fe(II)-Fenton system. If so, there might be a close relationship between the CP structures and their abilities to generate CL. As expected, similar ^●^OH-dependent intrinsic CL could also be generated from the other 18 CPs (Figure 3). The intensity of CL emission induced by CPs was strongly dependent on both chlorination level and chlorine substitution position. An obvious SAR between CPs structures and CL emission was observed [48,49,50]: (1) In general, as the chlorination level increases, the intensity of CL emission increases; (2) for CPs congeners, the CL increased in the order of para- < ortho- < meta-chlorine substitution with respect to the −OH group of CPs. For example, 2,5-dichlorophenol (DCP), 2,3,5-trichlorophenol (TCP) and 2,3,5,6-tetrachlorophenol (TeCP) generated the strongest CL emission among all the DCPs, TCPs and TeCPs congeners, respectively.

Actually, the properties that CL could be generated from all nineteen CPs in Fe(II)-Fenton system were also used to detect and quantify these ubiquitous CPs. As shown in Table 1, for those CPs that could produce an obvious CL emission, the LOD value could reach as low as 0.007 μM by this highly-sensitive CL analytical method.

**Figure 4:**
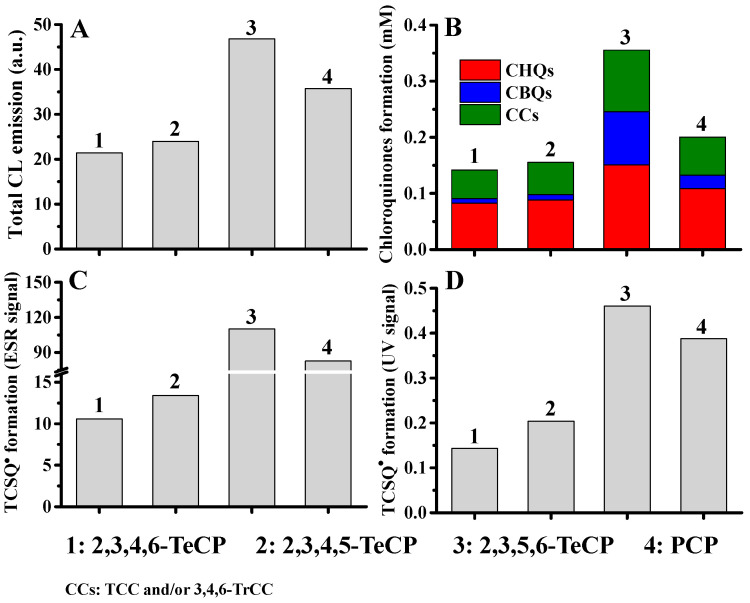
The intensity of CL emission from CPs correlated well with the total yields of the formation of corresponding chloroquinone intermediates and chlorosemiquinone radicals (taking PCP and three TeCPs for example) [50]. (**A**) The intensity of CL emission; (**B**) the total yields of CHQs/CBQs and CCs; (**C**,**D**) the yields of chlorosemiquinones measured by ESR (**C**) and UV−Vis method (**D**). The CL emission (**A**) was measured by an ultraweak CL analyzer, and the intensity of total CL emission was recorded. The maximum yields of chloroquinone intermediates (**B**) were measured by HPLC analysis. The maximum yields of CSQs^●^ were measured by both monitoring the ESR signal of CSQs^●^ through ESR analysis (**D**) and observing the typical UV−visible spectra of CSQs^●^ through UV−visible analysis. For detailed experimental procedure, please refer to our previous study [50]. For (**A**), CPs, 30 μM; H_2_O_2_, 100 mM; Fe(II)-EDTA, 1 mM. For (**B**), CPs, 1 mM; H_2_O_2_, 1 mM; Fe(II)-EDTA, 3 mM. For (**C**,**D**), CPs, 0.5 mM; H_2_O_2_, 0.5 mM; Fe(II)-EDTA, 1.5 mM. Copyright © 2016, with permission from Springer Nature. In the tests of acute toxicity to *Photobacterium phosphoreum*, a good relationship was observed between the chemical structures of 19 CPs and their acute toxicity [49,50]: (1) The higher the level of chlorine substitution, the stronger the toxicity of CPs; (2) for CPs congeners, their toxicity increased in the order of non- < mono- < di-ortho position-chlorophenols. Moreover, the rules for the relationship between the CPs structures and their degradation rates during ^●^OH-generating AOPs have been reported [56,57,58], which were listed as follows: (1) The higher the level of chlorine substitution, the slower the degradation rate of CPs; (2) for CPs congeners, the degradation rate decreased in the order of 3-/5- > 2-/4-/6-chlorine substitution CPs.

Similar to the degradation of PCP, the degradation of other CPs by Fenton system-mediated AOPs also generated chloroquinones as the major intermediates, they were chloro-1,4-benzoquinones (CBQs), chloro-1,4-hydroquinones (CHQs) and chloro-1,2-hydroquinones (also called chlorocatechols, CCs) [49,50]. Moreover, we have previously known that, all the above chloroquinones could react with H_2_O_2_ to produce CL, and the addition of Fenton reagent can markedly enhance CL emission [48]. In the studies on CL emission from all the nineteen CPs, CL emission of CPs was found to primarily depend on the yields and types of the corresponding chloroquinone intermediates generated from CPs [49,50]: a good relationship was observed between the CL intensity of CPs and the total yields of corresponding CBQs/CHQs and TCC/3,4,6-TCC (tetrachlorocatechol and 3,4,6-trichlorocatechol, the two CCs which emit stronger CL) (Figure 4A,B). More interestingly, not only chloroquinone intermediates, but chlorosemiquinone radicals (CSQs^●^) were also produced during the CL emission of CPs in Fenton-like systems, and the types and yields of which were also in good agreement with the emission of CL and the generation of chloroquinone intermediates (Figure 4).

In summary, based on the above results, together with the previously reported studies on SAR [56,57,59,60], we found good correlations between the CP structures and their chemical activities (CL emission, toxicity and degradation rate) as following listed [49,50]: (1) The higher the level of chlorine substitution for CPs, stronger CL emission, higher toxicity and slower degradation could be observed; (2) for CPs congeners, CPs with position-3 or -5 chlorine substitution show stronger CL emission, higher toxicity and faster degradation; while those CPs congeners with position-6 chlorine substitution show much weak CL emission, lower toxicity, and slower degradation. These findings may suggest that, utilizing the distinct CL-generating property of CPs induced by classic Fe(II)-Fenton system, a novel CL-based method can be developed, to not only detect and quantify trace amounts of CPs in pure or real samples, but also provide valuable information for evaluating the toxicity or degradation rate of CPs.

### 2.3. Similar to Chlorophenols, Other Classes of XAr Could Also Generate ^●^OH-Dependent Intrinsic CL Emission in the Degradation Mediated by Fe(II)-Fenton System

**Figure 5:**
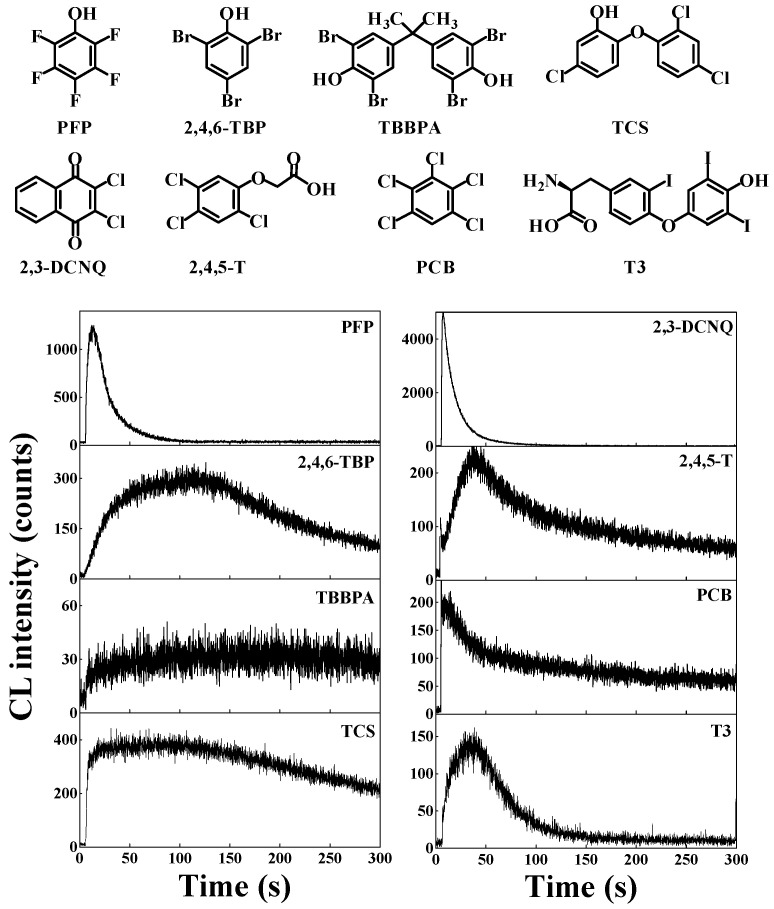
Similar ^●^OH-dependent intrinsic CL were also produced from the degradation of several typical XAr in classic Fe(II)-Fenton system [48]. The CL emission was measured by an ultraweak CL analyzer, with the CL signal recorded at a time interval of 0.1 s. PFP, 2,4,6-TBP, TBBPA, TCS, 2,4,5-T and PCB, 30 μM; 2,3-DCNQ, 30 µM; T3, 3 μM; Fe(II)–EDTA, 1 mM; H_2_O_2_, 100 mM. Copyright © 2015, American Chemical Society.

It should be noted that during the AOPs mediated by the ^●^OH-generating Fe(II)-Fenton system, besides PCP and the other eighteen chlorophenols, similar ^●^OH-dependent intrinsic CL was also generated from other halophenols and several other classes of XAr. These compounds include (Figure 5) [48]: other halophenols such as pentafluorophenol (PFP), 2,4,6-tribromophenol (2,4,6-TBP), the flame retardant 3,3′,5,5′-tetrabromobisphenol A (TBBPA), and the broad-spectrum antibacterial agent triclosan (TCS); halogenated naphthoquinone pesticides such as 2,3-dichloro-1,4-naphthoquinone (2,3-DCNQ); chlorophenoxyacetic acid herbicides such as the notorious 2,4,5-trichlorophenoxyacetic acid (2,4,5-T), one important component of Agent Orange; halogenated benzene biocides such as pentachlorobenzene (PCB); iodinated pharmaceuticals such as triiodothyronine (T3); These results indicate that most or even all XAr can generate ^●^OH-dependent CL in the degradation mediated by the Fe(II)-Fenton system. Moreover, similar CL spectra were also observed from these XAr, which were found to be attributed to the analogous molecular mechanism and similarity in structures of light-emitting species.

Similarly, based on the CL emission properties of XAr, we developed a novel and sensitive CL analytical method to detect and quantify these ubiquitous XAr. As anticipated, we successfully detected and quantified traces of several typical XAr, including PFP, 2,4,6-TBP, TBBPA, TCS, 2,3-DCNQ, 2,4,5-T, PCB and T3. For example, using this novel CL analytical method, we can directly detect concentrations as low as 0.03 μM for TCS, 0.07 μM for TBBPA, and 0.03 μM for T3 (Table 2) [48].

More importantly, this novel CL analytical method based on Fe(II)-Fenton system has been utilized to evaluate and detect whether XAr is contained in an actual environmental sample, the discharge from a paper mill [48]. As anticipated, obvious CL emission could be generated from the discharge of paper mill in the presence of Fenton agent, and further analysis suggested that the discharge contained 2,4-dichlorophenol and 4-chlorophenol. Furthermore, this newly-developed CL-based analytical method can also be used for monitoring the degradation kinetics of XAr in their treatment mediated by AOPs. In the PCP/Fe(II)-Fenton system, the kinetics of CL emission correlated well with the kinetics of PCP degradation [48]: the profiles of CL emission coincided with the kinetic curves of PCP degradation, and no further CL emission could be generated when PCP degradation finished.

## 3. Chemiluminescence-Based Analytical Methods Induced by Co(II)-Fenton-Like System for the Detection of XAr

### 3.1. Distinct Intrinsic CL Emission in the Degradation of Halohydroxyl Quinoid Compounds by Co(II)-Fenton-Like System: Markedly Different from the CL Produced by Classic Fe(II)-Fenton System

**Figure 6:**
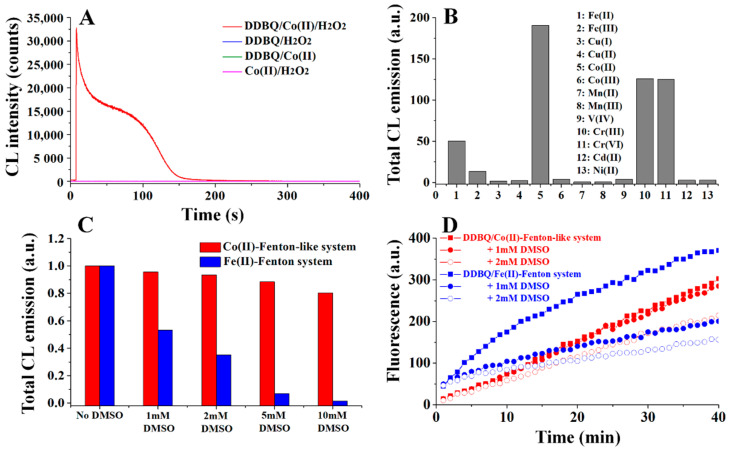
Distinct CL emission was generated from DDBQ in Co(II)-Fenton-like system, dependent on site specifically produced ^●^OH [51]. (**A**) The CL emission generated from DDBQ/Co(II)-Fenton-like system; (**B**) the intensity of CL emission generated by Co(II) was higher than other active transition metal ions; (**C**) DMSO only slightly inhibited CL emission from DDBQ in Co(II)-Fenton-like system, but significantly inhibited CL in Fe(II)-Fenton system; (**D**) the effect of DMSO on the generation of ^●^OH in DDBQ/Co(II)-Fenton-like system and DDBQ/Fe(II)-Fenton system. The CL emission (**A**–**C**) was measured by an ultraweak CL analyzer. Both the instant CL intensity and the intensity of total CL emission were recorded. (**D**) The generation of ^●^OH from Fenton or Fenton-like systems was measured through fluorescence analysis with terephthalic as the ^●^OH probe. For detailed experimental procedure, please refer to our previous study [51]. DDBQ, 10 µM; transition metal ions, 1 mM; H_2_O_2_, 100 mM. Copyright 2020, with permission from Elsevier.

As mentioned in the Introduction, unexpected ^●^OH-dependent intrinsic CL could be generated from TCBQ/H_2_O_2_, with DDBQ formed as an important final product, whereas neither ^●^OH nor CL could be generated by DDBQ and H_2_O_2_ [44]. However, it was unexpectedly discovered that a remarkable CL emission could be induced by adding some redox-active transition metal ions like Fe(II) and cobalt(II) (Co(II)), particularly for Co(II), which induced a much stronger CL emission than Fe(II) (Figure 6A,B) [51]. These results suggest that not only the key reactive oxygen species (ROS) intermediates for CL emission, but also the underlying molecular mechanism of the unexpected strong CL emission from DDBQ in Co(II)-Fenton-like system might be different from the CL in the classic Fe(II)-Fenton system.

**Figure 7:**
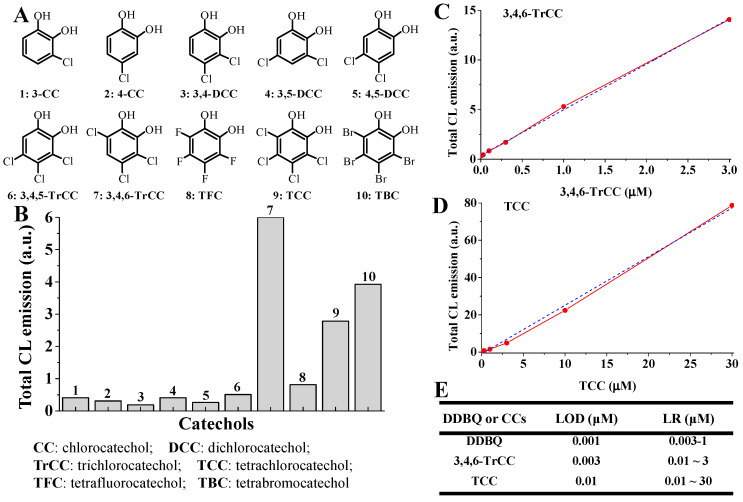
Analogous ^●^OH-dependent CL emission were also generated from halocatechols with the typical Co(II)-Fenton-like system, which can be utilized to quantify trace amount of halocatechols and DDBQ [51]. (**A**) The structure of several halocatechols; (**B**) the total CL intensity for these halocatechols; (**C**,**D**) CL emission of 3,4,6-TrCC and TCC; (**E**) the LOD and LR for the quantification of DDBQ, 3,4,6-TrCC and TCC by the distinct CL analytical method mediated by Co(II)-Fenton-like system. The CL emission was measured by an ultraweak CL analyzer, and both the instant CL intensity and the intensity of total CL emission were recorded. For (**B**), Halocatechols, 10 μM; Co(II), 0.05 mM; H_2_O_2_, 100 mM; For (**C**,**D**), Co(II), 0.5 mM; H_2_O_2_, 100 mM. Copyright 2020, with permission from Elsevier.

It should be noted that, in the Co(II)-Fenton-like system, similar CL emissions were also observed when substituting DDBQ with other halohydroxyl quinones such as 2,5-dibromo-3,6-dihydroxyl-1,4-benzoquinone, chlorocatechols such as 3-chlorocatechol, 3,4-dichlorocatechol, 3,4,6-trichlorocatechol, 3,4,5-trichlorocatechol and tetrachlorocatechol (the latter two are typical effluents from bleached kraft pulp mills [61]), and other halocatechols such as tetrabromocatechol and tetrafluorocatechol (Figure 7A,B) [51].

### 3.2. Site Specifically Produced ^●^OH, but Not Free ^●^OH Is Responsible for the CL Production of Halohydroxyl Quinoid Compounds Induced by Co(II)-Fenton-Like System

We have previously known that the generation of free ^●^OH was critical for CL generation by TCBQ (the precursor of DDBQ) and H_2_O_2_, and adding ^●^OH scavenger could markedly inhibit CL emission [44]. However, we found it is not the case for the CL induced by DDBQ in Co(II)-Fenton-like system: CL emission was slightly inhibited by adding DMSO (Figure 6C). This strongly indicates that free ^●^OH is not responsible for the CL emission induced by DDBQ in the Co(II)-Fenton like system.

However, although free ^●^OH may not be involved in CL emission from the DDBQ/Co(II)-Fenton-like system, ^●^OH generation was also detected and confirmed [51]. A relatively weak ESR signal of DMPO/^●^OH was observed from the CL reaction of DDBQ with the Co(II)-Fenton-like system, and adding DMSO could diminish the ESR signal of DMPO/^●^OH with the concurrent generation of the secondary radical DMPO/^●^CH_3_, indicating ^●^OH was indeed produced. It should be noted that the signal of secondary radical DMPO/^●^CH_3_ was still relative weak even after the added DMSO completely diminished the signal of DMPO/^●^OH. However, for ^●^OH production in the CL reaction of DDBQ with the classic free ^●^OH-generating Fe(II)-Fenton system, the DMPO/^●^OH signal could be markedly diminished by adding DMSO, with the concurrent generation of the strong secondary radical DMPO/^●^CH_3_. In addition, for the kinetics of ^●^OH formation, adding DMSO only partly inhibited ^●^OH generation from the CL reaction of DDBQ in the Co(II)-Fenton-like system, whereas it markedly inhibited ^●^OH generation in the classic Fe(II)-Fenton system (Figure 6C,D). These results suggest that for the CL reaction of DDBQ with the Co(II)-Fenton-like system, ^●^OH might be generated as the site specifically produced •OH, but not the free ^●^OH.

Interestingly, besides the site specifically produced ^●^OH, other ROS, such as O_2_^●−^ and ^1^O_2_, were also generated from the CL reaction of DDBQ with the Co(II)-Fenton-like system [51]. The ROS intermediates from this distinct CL reaction of DDBQ with the Co(II)-Fenton-like system was significantly distinct from the CL reaction by TCBQ/H_2_O_2_ and the CL reaction induced by the classic Fe(II)-Fenton system mentioned above. Based on these results, we further varied the molar ratio of DDBQ:Co(II) to investigate the correlations between ROS production and CL emission, and to confirm which ROS is crucial for the generation of CL emission. As expected, not only the CL emission, but also the types and yields of ROS were significantly affected by changing the ratio of DDBQ:Co(II).

For the role of O_2_^●−^, it seems that O_2_^●−^ is not responsible for the CL generation from DDBQ in Co(II)-Fenton-like system [51]. O_2_^●−^ generation was hardly affected by changing the ratio of DDBQ:Co(II), suggesting that O_2_^●−^ is not the crucial ROS responsible for CL emission. Moreover, we also found that ^1^O_2_ is not critical for initiating CL emission [51]. Although ^1^O_2_ production could be enhanced by adding DDBQ, and large quantity of ^1^O_2_ can be produced, most ^1^O_2_ was generated only in the later stage. No matter how the ratio of DDBQ:Co(II) was varied, almost no ^1^O_2_ was generated in the early stage, whereas CL emission had been generated in this stage.

However, for the role of ^●^OH, a close relationship between ^●^OH generation and DDBQ degradation was clearly observed: the higher the concentration of DDBQ, the lower the yields of ^●^OH generation. Moreover, the degradation of DDBQ also correlated well with CL emission: the process of generating CL emission was accompanied by the degradation of DDBQ, and when DDBQ degradation achieved the maximum, no further CL emission can be observed. These results indicate that CL emission is closely related to and probably dependent on the generation of site specifically produced ^●^OH. More interestingly, different from the results observed in the classic Fe(II)-Fenton system, the inhibitory effect of DMSO on the kinetics of both CL emission and ^●^OH generation were relatively weak in Co(II)-Fenton-like system (Figure 6C,D). These results further confirmed that CL generation from the DDBQ/Co(II)-Fenton-like system is indeed dependent on site specifically produced ^●^OH, but not free ^●^OH, O_2_^●−^ and ^1^O_2_ [51]. In other words, the site specifically produced ^●^OH might be the initiating ROS for CL emission.

### 3.3. The Molecular Mechanism for the Site-Specific ^●^OH-Dependent CL Emission of Halohydroxyl Quinoid Compounds in Co(II)-Fenton-Like System

Then the questions are how to generate site-specific ^●^OH and how to induce the intrinsic CL emission of halohydroxyl quinoid compounds such as DDBQ? The further investigation indicated that during the CL reaction of DDBQ with Co(II)-Fenton-like system, Co(II) could combine with DDBQ to form a Co(II)-DDBQ complex through the active bidentate coordinate sites -C(O)C(OH) [62,63], and then the attack by H_2_O_2_ may result in the generation of site-specific ^●^OH at the coordinates sites, which then induce the degradation of DDBQ and the concurrent CL emission [51].

**Scheme 2:**
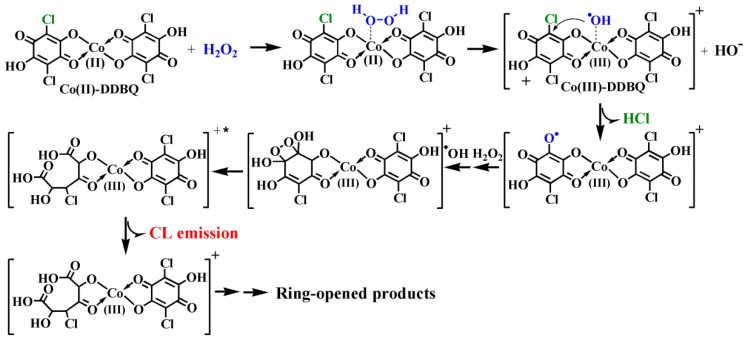
The potential molecular mechanism for the unexpectedly strong CL emission from DDBQ/Co(II)-Fenton-like system mainly depends on site specifically produced ^●^OH [51]. Copyright 2020, with permission from Elsevier.

In order to better clarify the molecular mechanism for the distinct CL emission from DDBQ in the Co(II)-Fenton like system, the conversion of Co(II) was investigated in detail. As anticipated, we further found that Co(II) was transformed to Co(III) on the basis of UV−Vis analysis on the production of the Co(III)-complex [64] and the XPS analysis [65,66] on the binding energy of Co for the samples before or after reaction. On the basis of all the above findings and our previous results, we proposed the underlying molecular mechanism for the distinct site-specific ^●^OH-dependent CL emission from the degradation of DDBQ mediated by Co(II)-Fenton-like system (Scheme 2) [51]:

### 3.4. Highly-Sensitive CL-Based Analytical Method for the Detection of Halohydroxyl Quinoid Compounds on the Basis of Co(II)-Fenton-Like System

**Figure 8:**
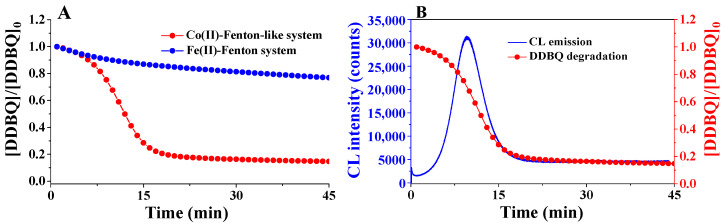
(**A**) The degradation of DDBQ in Co(II)-Fenton-like system was more effective than that in the classic Fe(II)-Fenton system, and (**B**) good correlation can be observed between CL emission and DDBQ degradation in Co(II)-Fenton-like system [51]. The kinetics of DDBQ degradation (**A,B**) was monitored by HPLC analysis. The CL emission (**B**) was measured by an ultraweak CL analyzer, with the CL signal recorded at a time interval of 0.1s. DDBQ, 3 mM; transition metal ions, 3 mM; H_2_O_2_, 300 mM. Copyright 2020, with permission from Elsevier.

As mentioned above, the CL intensity of DDBQ in Co(II)-Fenton-like system is markedly stronger than that in classic Fe(II)-Fenton system. Therefore, this distinct CL-generating property of DDBQ induced by Co(II)-Fenton-like system might be utilized to measure and quantify trace amounts of DDBQ, and to monitor the degradation of DDBQ as well. Indeed, by using this novel CL-based analytical method, the LR for the quantitative detection of DDBQ is 3–1000 nM, and the LOD value is as low as 1 nM [51], which is much lower than the LOD value (0.5 μM) of DDBQ by using the reported traditional HPLC method. Compared with the traditional analytical methods, such as HPLC [67] on the detection of DDBQ, this novel CL-based analytical method exhibits a series of advantages, such as being relatively simple, rapid, sensitive, and without a complicated pretreatment process. Moreover, the kinetics of CL emission was in good agreement with the kinetics of DDBQ degradation: CL can be produced during DDBQ degradation, and when DDBQ was completely degraded, CL emission was no longer observed (Figure 8B). Interestingly, we found that the efficiency for DDBQ degradation mediated by the Co(II)-Fenton-like system was much higher than the classic Fe(II)-Fenton system (Figure 8A) [51], possibly due to the site-specific ^●^OH, which can be effectively used to degrade adjacent DDBQ, that was produced in the former system, whereas free ^●^OH was produced in the latter system. These results indicate that in the treatment or degradation of those pollutants with structures containing bidentate coordination sites that can typically bind with transition metal ions, the technology consisting of AOPs mediated by the Co(II)-Fenton-like system may degrade target pollutants more effectively, because the degradation occurring site specifically exhibits a higher efficiency.

More importantly, as mentioned in Section 3.1., in the Co(II)-Fenton-like system, typically strong CL emission can be produced from halocatechols. All the above halocatechols have a similar bidentate coordinated site(-C(OH)C(OH)), which can well coordinate with transition metal ions such as Co(II). Similarly, compared with Fe(II)-Fenton system, these halocatechols in the Co(II)-Fenton-like system produced significantly stronger CL emissions. Interestingly, the distinct CL analytical method based on the Co(II)-Fenton-like system can also be applied to detect and quantify halocatechols. By utilizing this unique CL-based method, the LOD values for 3,4,6-trichlorocatechol and tetrachlorocatechol can reach as low as 3 nM and 10 nM, respectively, [51] (Figure 7C–E). These results, together with above findings on the measurement of DDBQ, indicate that this novel CL analytical method based on the Co(II)-Fenton-like system can be applied to detect and quantify DDBQ (or its analogues), halocatechols and other halohydroxyl quinoid compounds.

## 4. The Advantages and Challenges of the Typical CL-Based Analytic Methods for the Detection of XAr in their Environmental Applications

Compared with the traditional methods for the detection and quantification of XAr, the novel CL-based analytical method mediated by the Fe(II)-Fenton or Co(II)-Fenton-like system displayed a series of advantages: (1) It is relatively selective. Obvious CL emission can be produced from samples containing XAr, whereas no CL can be observed from samples that do not contain XAr. (2) It is extremely sensitive. Both the LOD and LR values for the quantification of XAr by this CL-based method are lower than the values obtained from traditional analytical methods. (3) It is simple, fast and low-cost. This CL-based method is easy to operate, without expensive apparatus and complicated pretreatment.

However, this CL-based analytical method for the detection of XAr also faces potential challenges during its environmental applications. It can only evaluate whether a sample contains XAr, but cannot accurately identify the specific kind of XAr. In order to unambiguously identify the XAr contained in the sample, the CL-based method needs to be combined with other qualitatively analytical methods. Moreover, the organic pollutants that can be detected by this novel CL analytical method are limited: it can detect and measure only those halocompounds with aromatic structures, but not the non-halogenated aromatic compounds and haloaliphatic compounds. Therefore, it is necessary to develop a more efficient and sensitive CL-producing system based on this typical CL analytical method of XAr, to detect and quantify more environment pollutants. These would be addressed in our future studies.

## 5. Conclusions

On the basis of the above series of studies, we have made important progress in the research of CL emission generated from the degradation of ubiquitous XAr mediated by AOPs, which have broad chemical and environmental implications.

We found that the degradation of highly-toxic PCP in AOPs mediated by the classic Fe(II)-Fenton system can unprecedentedly produce intrinsic CL emission, specifically dependent on the generation of free ^●^OH. Interestingly, besides PCP, all nineteen chlorophenols can be induced to generate ^●^OH-dependent intrinsic CL by the classic Fe(II)-Fenton system, and the underlying SAR for CL of these chlorophenols was revealed: (1) In general, the CL emission increased with the increase of chlorination level; (2) for CP congeners, the CL emission decreased in the following order of 3-/5- > 2-/4-/6-chlorine substitution CPs; (3) the CL intensity for each CP was determined by the types and the total yields of corresponding chloroquinone intermediates and semiquinone radicals. Additionally, several kinds of XAr, including the broad-spectrum antibacterial agent triclosan, the flame retardant TBBPA, the widely used herbicides 2,4,5-T, and iodinated pharmaceuticals T3, were capable of generating similar ^●^OH-dependent CL. Based on these results, a novel and sensitive CL analytical method was developed, which can not only detect and quantify these ubiquitous XAr, but also monitor their degradation kinetics, and provide useful information for evaluating their toxicity to organisms and degradation rate.

However, for those XAr with structures containing the group of bidentate coordination sites, such as halohydroxyl quinone and halocatechol, the degradation of which in AOPs mediated by the distinct Co(II)-Fenton-like system would generate significant CL emission, much stronger than that generated in the classic Fe(II)-Fenton system, which was found to be attributed to the site-specific generation of reactive ^●^OH. Consequently, a more sensitive CL analytical method via the Co(II)-Fenton-like system was developed to detect and quantify halohydroxyl quinoid compounds or those compounds with similar structures, and monitor their degradation kinetics. Moreover, the AOPs consisting of the typical Co(II)-Fenton-like system can be effectively used to selectively degrade and treat halohydroxyl quinoid compounds, and this is because these compounds can bind with Co(II) to site-specifically produce the highly-reactive ^●^OH, which can be effectively used to degrade the target pollutants.

## Figures and Tables

**Table 1:** The limit of detection (LOD) and linear range (LR) for the quantification of CPs by the novel CL-based analytical method mediated by Fe(II)-Fenton system [49,50]. Copyright © 2017, American Chemical Society. Copyright © 2016, with permission from Springer Nature.

CPs	LOD (μM)	LR (μM)
2,3,4-TCP	0.3	0.3~100
2,4,6-TCP	0.3	0.3~100
3,4,5-TCP	0.07	0.1~100
2,4,5-TCP	0.01	0.03~100
2,3,6-TCP	0.07	0.07~100
2,3,5-TCP	0.003	0.007~100
2,3,4,6-TeCP	0.01	0.03~100
2,3,4,5-TeCP	0.01	0.03~100
2,3,5,6-TeCP	0.007	0.01~100
PCP	0.007	0.01~100

**Table 2:** The limit of detection (LOD) and linear range (LR) for the quantification of CPs by the novel CL-based analytical method mediated by Fe(II)-Fenton system [48]. Copyright © 2015, American Chemical Society.

XAr	LOD (μM)	LR (μM)
PCP	0.01~70	0.007
TCS	0.07~30	0.03
TBBPA	0.1~10	0.07
2,3-DCNQ	0.1~100	0.07
PCB	0.1~10	0.07
PCB	0.1~10	0.07
T3	0.03~1	0.03

## Data Availability

Not applicable.
